# Use of benzodiazepines is the risk factor for infection in patients aged 80 years or older with diffuse large B-cell lymphoma: A single-institution retrospective study

**DOI:** 10.1371/journal.pone.0269362

**Published:** 2022-06-10

**Authors:** Anna Ogiso, Tomohiro Mizuno, Kaori Ito, Fumihiro Mizokami, Akihiro Tomita, Shigeki Yamada

**Affiliations:** 1 Department of Clinical Pharmacy, Fujita Health University School of Medicine, Toyoake, Japan; 2 Department of Hematology, Fujita Health University School of Medicine, Toyoake, Japan; 3 Department of Pharmacy, National Center for Geriatrics and Gerontology, Obu, Japan; European Institute of Oncology, ITALY

## Abstract

**Background:**

The number of patients aged 80 years or older with diffuse large B-cell lymphoma (DLBCL) is increasing, and the incidence rate of the disease in this population group reaches up to 20%. The risk of infection is higher in older patients than in other patients. Although hypnotic drugs are frequently detected as potentially inappropriate medications, it is unclear whether hypnotic drugs affect the occurrence of infection during chemotherapy. Here, we investigated whether the use of hypnotic drugs is associated with infection during first-line chemotherapy in patients with diffuse large B-cell lymphoma (DLBCL) aged 80 years or older.

**Methods:**

Japanese patients aged 80 years or older with diffuse large B-cell lymphoma who had received first-line chemotherapy at Fujita Health University Hospital from January 2005 to March 2020 were enrolled in this retrospective cohort study. The primary study outcome was the identification of the risk factor for infection during first-line chemotherapy.

**Results:**

This study included 65 patients received first-line chemotherapy. The proportion of patients with National Comprehensive Cancer Network-international prognostic index ≥ 6 was higher in the infection group than in the non-infection group. The relative dose intensity of each anticancer drug (cyclophosphamide, adriamycin, and vincristine) and dose of prednisolone did not significantly differ between the two groups. Multivariate analysis showed that the use of benzodiazepines was a risk factor for infection (odds ratio, 4.131 [95% confidence interval: 1.225–13.94], *P* = 0.022).

**Conclusion:**

DLBCL patients using benzodiazepines should be monitored for infection symptoms during chemotherapy.

## Introduction

Diffuse large B-cell lymphoma (DLBCL) is the most frequent type in non-Hodgkin lymphoma (NHL) [1, 2]. In Japan, DLBCL has been reported to account for 45.3% of all cases of NHL [3]. The number of patients aged 80 years or older with DLBCL is increasing [4], and the frequency of those patients reaches approximately 20% [3]. Older patients with DLBCL show variable degrees of functional impairment, comorbidity, chronic undernutrition, and altered drug metabolism [5, 6]. Therefore, the risk of adverse drug reactions is higher in older patients than in other patients. To improve treatment outcomes and avoid severe adverse drug reactions, the type of chemotherapeutic regimen and dose intensity are determined considering the National Comprehensive Cancer Network (NCCN)-international prognostic index (IPI) for the prediction of outcomes in patients with aggressive lymphoma [7]. The NCCN-IPI helps predict mortality in patients with DLBCL by using age, lactate dehydrogenase (LDH) levels, Ann Arbor stage, and Eastern Cooperative Oncology Group performance status (PS). Although the NCCN-IPI is a good predictor of mortality, it remains unclear whether it can be a predictor of adverse drug reactions during chemotherapy.

An infectious episode is a well-known adverse drug reaction and has been reported to be associated with increased mortality [8, 9]. Comorbidity is reported as an independent risk factor for severe adverse drug reactions [10]. In addition, older age [11] and rituximab (R)-based chemotherapy [12] increase the risk of infection. Patients aged 75 years or older are classified as the group with the highest risk of poor prognoses per the NCCN-IPI and account for 40.8% of all patients with DLBCL [3]. Thus, the identification of risk factors for infection might improve clinical outcomes in such older patients (e.g., patients aged 80 years or older).

Inappropriate medication use and drug interactions are resulting from poor functional impairment in older patients. Several screening tools are available to evaluate the appropriateness of pharmacotherapy for older patients and to avoid inappropriate medication use and drug interactions. Although these tools have shown that hypnotic drugs increase the risk for falls or fractures [13, 14], it is unclear whether hypnotic drugs influence the occurrence of infection during chemotherapy. Hence, in this study, we investigated whether the use of hypnotic drugs is associated with infection during first-line chemotherapy in patients aged 80 years or older.

## Materials and methods

### Study design and data source

Japanese patients aged 80 years or older with DLBCL who had received first-line chemotherapy at Fujita Health University Hospital from January 2005 to March 2020 were enrolled in this retrospective cohort study. All medical data were collected from the hospital’s medical records. The exclusion criteria were as follows: (a) patients whose baseline data were unavailable and (b) patients without completed first-line chemotherapy. The standard chemotherapy regimens used were R-CHOP (a combination of R, cyclophosphamide [CPA], adriamycin [ADR], vincristine [VCR], and prednisolone [PSL]), CHOP (a combination of CPA, ADR, VCR, and PSL), COP (a combination of CPA, VCR, and PSL), R-COP (a combination of R, CPA, VCR, and PSL), CHP (a combination of CPA, ADR, and PSL), R-CHP (a combination of R, CPA, ADR, and PSL), and R-HOP (a combination of R, ADR, VCR, and PSL). The RDI was calculated by dividing the DI of each chemotherapeutic drug (R, CPA, ADR, and VCR) by the respective target DI and multiplying by 100 [15]. Disorders were categorized according to the modified Charlson Comorbidity Index (CCI) [6, 16].

### Outcome measures

The identification of the risk factors for infection during first-line chemotherapy was the primary outcome of this study. Infection severity was defined in terms of grade 2 or higher based on the Common Terminology Criteria for Adverse Events version 5.0, as follows:

- Grade 2: Oral intervention indicated (e.g., antibiotic, antifungal, or antiviral)
- Grade 3: IV antibiotic, antifungal, or antiviral intervention indicated; invasive intervention indicated
- Grade 4: Life-threatening consequences; urgent intervention indicated
- Grade 5: Death

Patients received administration of prophylactic antibiotics were not defined as CTCAE grade 2.

The NCCN-IPI was determined as described in a previous report [7]. Briefly, age, lactate dehydrogenase ratio (ratio to the institutional upper limit of normal), Ann Arbor stage, the presence of extranodal disease, and PS were used to determine the NCCN-IPI.

### Statistical analyses

All data are shown as medians and ranges. The Mann–Whitney U test was used for comparisons of data without normal distribution. The chi-square test and Fisher’s exact test were used to analyze nominal scales. Logistic regression analysis using a multivariable model was performed to identify the risk factors for the infection events. To determine the risk factors for infection, logistic regression analysis with a multivariable model was applied for the following covariates: NCCN-IPI and benzodiazepine use. To avoid multicollinearity with the NCCN-IPI, PS was not included in the multivariate analysis. The fitness of the logistic regression model was evaluated using the Hosmer-Lemeshow test. The predictive ability was evaluated by plotting the receiver operating characteristic curve. A two-sided *P*-value of <0.05 was considered significant in all statistical analyses, which were performed using SPSS version 22.0 (SPSS Inc., Chicago, IL, USA).

### Ethics approval

This study was approved by the ethics board of Fujita Health University Hospital (ethics committee approval number: HM21-205) and conducted according to the appropriate guidelines. Because this was a retrospective cohort study, an opt-out approach for informed consent was used according to the approval of the ethics board.

## Results

### Patient characteristics

**Fig 1:**
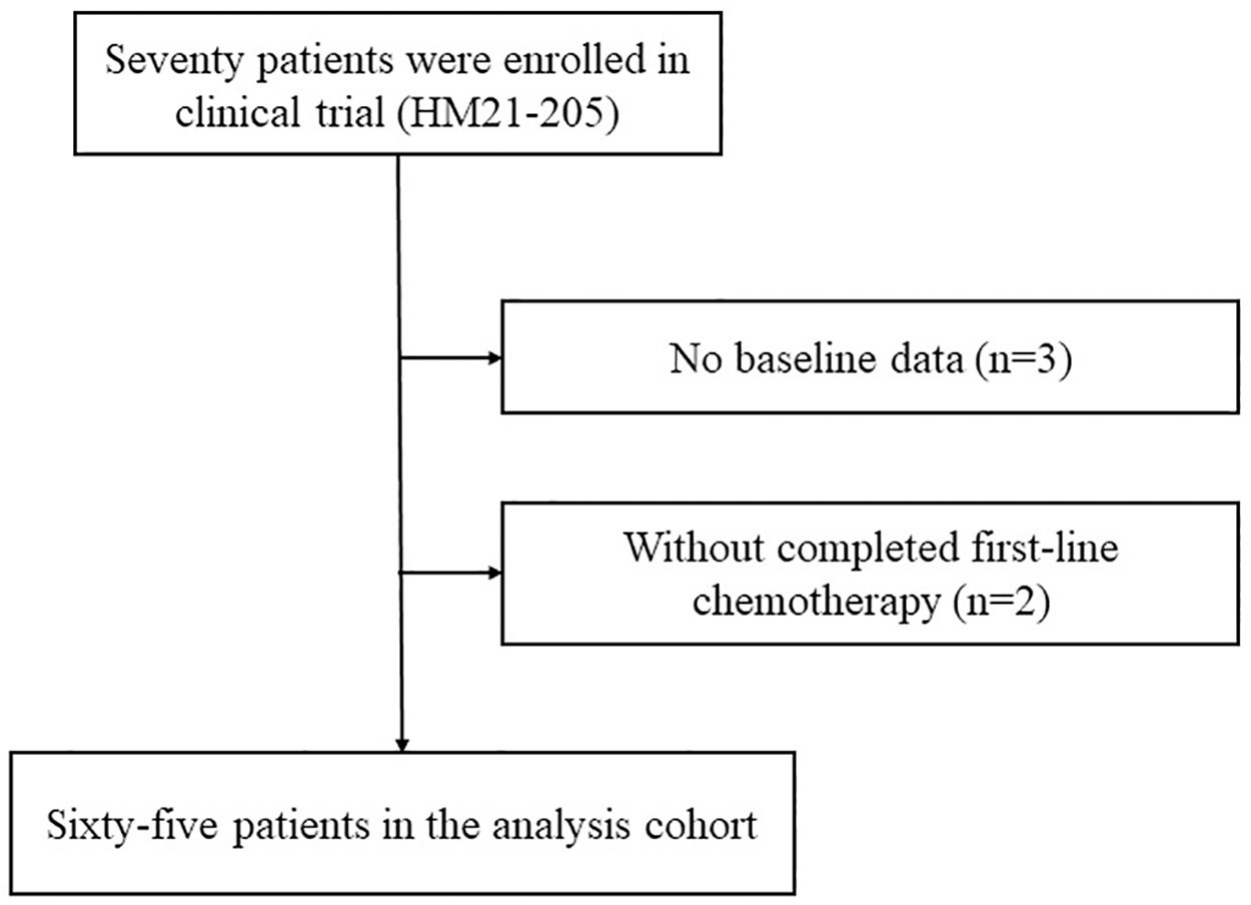
Study protocol.

This study included 65 patients who had received first-line chemotherapy at Fujita Health University Hospital (Fig 1). The baseline characteristics of the subjects are shown in Table 1. The median age was 83 years in the all subjects. The CCI for each patient ranged from 2 to 9. Patients with or without infection events were divided into non-infection (n = 46) and infection (n = 19) groups, respectively. The PS in the infection group was significantly higher than that in the non-infection group (*P* = 0.049). The proportion of patients with NCCN-IPI of ≥6 was higher in the infection group than in the non-infection group (*P* = 0.053). The RDI of each drug (R, CPA, ADR, and VCR) and PSL dose were not significantly different between the two groups. Furthermore, the number of concomitant medications was not significantly different between the groups (Table 1), and the types of concomitant medications were similar in the two groups (Table 2). However, the proportion of patients using benzodiazepines was higher in the infection group than in the non-infection group (Table 2).

**Table 1:** Baseline characteristics.

Baseline Characteristics	Total (n = 65)	Infection group (n = 19)	Non-infection group (n = 46)	*P* value
Age, yrs (range)	83.0 (80–91)	82.5 (80–90)	83.0 (80–91)	0.550
Male, no (%)	23 (35.4)	6 (31.6)	17 (37.0)	0.780^a^
Body surface area (range)	1.43 (1.06–1.67)	1.45 (1.06–1.66)	1.43 (1.15–1.67)	0.988
Body mass index (range)	20.4 (15.5–33.4)	21.5 (16.4–31.0)	20.3 (15.5–33.4)	0.306
Performance status (range)	2 (0–4)	2 (1–4)	1 (0–4)	0.049
≥ 2, no (%)	36 (55.4)	14 (73.7)	22 (47.8)	0.056^b^
Extranodal disease, no (%)	23 (35.4)	7 (36.8)	16 (34.8)	1.000^a^
Bone marrow infiltration, no (%)	9 (13.8)	2 (10.5)	7 (15.2)	1.000^a^
Central nervous system infiltration, no (%)	1 (1.53)	1 (5.26)	0 (0)	0.292^a^
Ann Arbor stage III-IV, no (%)	35 (53.8)	12 (63.2)	24 (52.2)	0.418^b^
Number of neutrophil, 10^^3^/μL (range)	3.85 (0.2.8–125)	4.08 (2.77–12.5)	3.74 (0.28–10.9)	0.289
Number of platelet, 10^^4^/μL (range)	19.9 (3.4–43.8)	19.6 (3.4–32.2)	20.1 (10.1–43.8)	0.466
Albumine, g/dL (range)	3.4 (1.8–5.2)	3.5 (1.8–4.1)	3.4 (2.1–5.2)	0.795
LDH ratio (range)	1.14 (0.60–13.6)	1.41 (0.73–13.5)	1.11 (0.60–4.84)	0.489
0, no (%)	26 (40.0)	7 (36.8)	19 (41.3)	0.433^b^
1, no (%)	31 (47.7)	10 (52.6)	21 (45.7)	
2, no (%)	8 (12.3)	2 (10.5)	6 (13.0)	
NCCN-IPI	5 (3–8)	6 (3–7)	5 (3–8)	0.219
≥ 6, no (%)	29 (44.6)	12 (63.2)	17 (37.0)	0.053^b^
CCI	3 (2–9)	2 (2–4)	3 (2–9)	0.245
≥ 3, no (%)	34 (52.3)	8 (42.1)	26 (56.5)	0.413^a^
Number of concomitant medications	5 (0–15)	7 (0–12)	5 (0–15)	0.417
≥ 6 medications, no (%)	29 (44.6)	11 (57.9)	18 (39.1)	0.166^b^
Chemotherapy regimen and RDI				
R-CHOP, no (%)	21 (32.3)	9 (47.4)	12 (26.1)	0.642^b^
CHOP, no (%)	27 (41.5)	6 (31.6)	21 (45.7)	
COP, no (%)	5 (7.69)	2 (10.5)	3 (6.52)	
R-COP, no (%)	9 (13.8)	2 (10.5)	7 (15.2)	
CHP, no (%)	1 (1.54)	0 (0.00)	1 (2.17)	
R-CHP, no (%)	1 (1.54)	0 (0.00)	1 (2.17)	
R-HOP, no (%)	1 (1.54)	0 (0.00)	1 (2.17)	
Rituximab RDI, % (range)	98.4 (0–106)	97.5 (0–105)	99.2 (0–106)	0.264
Cyclophosphamide RDI, % (range)	69.2 (0–83.3)	65.2 (49.5–80.3)	68.5 (0–83.3)	0.863
Doxorubicin RDI, % (range)	59.9 (0–83.3)	61.3 (0–77.7)	59.6 (0–83.3)	0.873
Vincristine RDI, % (range)	68.4 (0–97.1)	66.9 (42.9–71.7)	68.4 (0–97.1)	0.920
Prednisolone, mg (range)	50 (0–100)	50 (0–60)	52 (0–100)	0.188

CCI: Charlson Comorbidity Index, LDH: lactate dehydrogenase, NCCN-IPI: National Comprehensive Cancer Network-international prognostic index, R: rituximab, C: cyclophosphamide, H: adriamycin, O: vincristine, P: prednisolone, RDI: relative dose intensity,

^a^Fisher’s exact test,

^b^chi-square test

**Table 2:** Types of concomitant medications.

Types of concomitant medications	Infection group (n = 19)	Non-infection group (n = 46)	*P* value
Antihypertensive drugs (n) (%)	15 (78.9)	29 (63.0)	0.212^b^
Antidiabetic drug (n) (%)	0 (0)	6 (13.0)	0.169^a^
Lipid-lowering drugs (n) (%)	7 (36.8)	17 (37.0)	1.000^a^
Anticoagulant or antiplatelet drugs (n) (%)	6 (31.6)	15 (32.6)	1.000^a^
Antiulcer drug (n) (%)	9 (47.4)	19 (41.3)	0.784^a^
Hypnotic drug (n) (%)	10 (52.6)	8 (17.4)	0.006^a^
• Benzodiazepines (n) (%)	9 (47.3)	8 (17.4)	0.027^a^
Anti-osteoporosis drug (n) (%)	3 (15.8)	10 (21.7)	0.740^a^

^a^Fisher’s exact test,

^b^chi-square test

### Risk factors for infection

**Fig 2:**
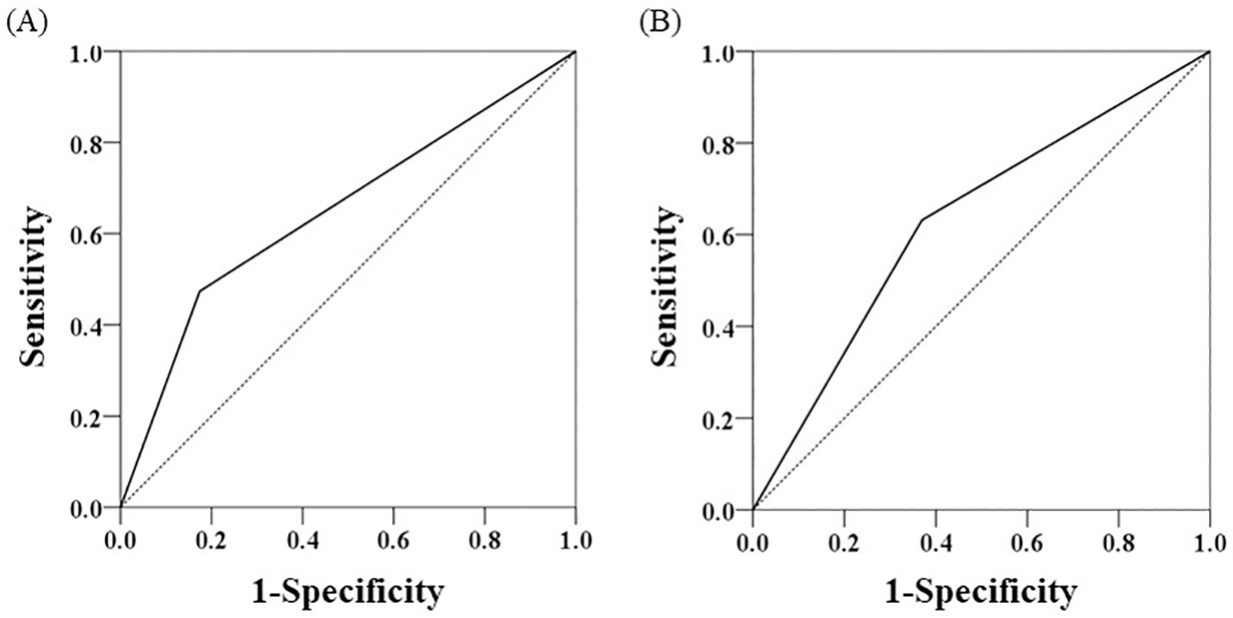
Receiver operating characteristic curve analysis of the occurrence of infection. (A) Receiver operating characteristic (ROC) analysis based on the use of benzodiazepines. Sensitivity, 0.474; specificity, 0.826; AUC, 0.650 (95% CI, 0.495–0.805), *P* = 0.059. (B) ROC analysis based on NCCN-IPI of ≥6. Sensitivity, 0.632; specificity, 0.632; AUC, 0.631 (95% CI, 0.481–0.781), *P* = 0.099. AUC, area under the curve; CI, confidence interval.

The results of univariate and multivariate analyses are shown in Table 3. The univariate analysis revealed that poor PS and the use of benzodiazepines were significant risk factors for infection (PS ≥ 2, OR = 3.055 [95% CI: 0.945–9.877], *P* = 0.056; NCCN-IPI ≥ 6, OR = 2.924 [95% CI: 0.966–8.855], *P* = 0.053; use of benzodiazepines, OR = 4.275 [95% CI: 1.314–13.91], *P* = 0.027). Meanwhile, extranodal disease, bone marrow infiltration, central nervous system infiltration, advanced cancer, CCI, polypharmacy, and the use of antiulcer drug were not associated with infection events. NCCN-IPI affects clinical outcomes more than PS, and these are confounding factors. Therefore, we performed multivariate analysis using NCCN-IPI and use of benzodiazepines. The use of benzodiazepines was identified as a risk factor for infection in multivariate analysis (OR = 4.131 [95% CI: 1.225–13.94], *P* = 0.022). To determine whether the use of benzodiazepines is a predictor of the occurrence of infection, we evaluated the predictive ability by using receiver operating characteristic curve analysis. The use of benzodiazepines showed a trend toward good prediction of the occurrence of infection (sensitivity, 0.474; specificity, 0.826; AUC, 0.650 [95% CI, 0.495–0.805], *P* = 0.059) (Fig 2).

**Table 3:** Univariate and multivariate analyses of the risk factors for infection.

Characteristics	Univariate analysis	*P* value	Multivariate analysis	*P* value
	OR (95%CI)		OR (95%CI)	
PS ≥ 2	3.055 (0.945–9.877)	0.056		
Extranodal disease	1.094 (0.360–3.326)	1.000		
Bone marrow infiltration	0.655 (0.123–3.487)	1.000		
Central nervous system infiltration	1.056 (0.949–1.174)	0.292		
Ann Arbor stage III-IV	1.571 (0.525–4.707)	0.418		
NCCN-IPI ≥ 6	2.924 (0.966–8.855)	0.053	2.811 (0.881–8.969)	0.081
CCI ≥ 3	0.559 (0.190–1.650)	0.413		
Number of concomitant medication ≥ 6	2.139 (0.722–6.338)	0.166		
Antiulcer drug	1.279 (0.437–3.747)	0.784		
Benzodiazepine use	4.275 (1.314–13.91)	0.027	4.131 (1.225–13.94)	0.022

OR: odds ratio; CI: confidence interval.

### Types of infections

Types of infections and pathogens are shown in Table 4. The presence of infection was determined by hematology clinicians. Based on the Common Terminology Criteria for Adverse Events version 5.0 grade of infection, any use of oral or IV antibiotics was considered infection. Febrile neutropenia was the most frequent infection (26.3%, n = 5), followed by the common cold (15.7%, n = 3). Five patients (26.3%) in the infection group had oral (gingival lesion, periodontitis) and gastrointestinal (including bile duct) infections.

**Table 4:** Types of infections and pathogen.

Types of Infections	Pathogen (n)	No (%)
Febrile neutropenia	Unknown(5)	5 (26.3)
Elevated C-reactive protein level and having common cold symptoms	Unknown(3)	3 (15.7)
Bacterial septicemia	*Escherichia coli*(1) MRSE(1)	2 (10.5)
Infection from gastrointestinal tract lesion	*Enterococcus* sp.(1) Unknown(1)	2 (10.5)
CRBSI	Unknown (1)	1 (5.2)
Cholangitis	*Enterococcus faecium* (1)	1 (5.2)
Urinary tract infection	Unknown(1)	1 (5.2)
Infection from gingival lesion	*Corynebacterium* sp (1)	1 (5.2)
Herpes simplex	Herpes simplex virus type 1 (1)	1 (5.2)
Periodontitis	Unknown(1)	1 (5.2)
Infection from skin inflammation	MSSA (1)	1 (5.2)

CRBSI: catheter-related blood stream infection

MRSE: *Methicillin-Resistant Staphylococcus epidermidis*

MSSA: *meticillin-susceptible S*. *aureus*

## Discussion

This study was aimed at investigating whether the use of hypnotic drugs is associated with infection during first-line chemotherapy in patients with DLBCL aged 80 years or older. Our results suggested that compared to a high NCCN-IPI, the use of benzodiazepines was potentially associated with infection events during chemotherapy. Although dose intensity is related to mortality in patients with DLBCL [17], it was not identified as a risk factor for infection in our study.

Older patients are at a higher risk of infection-related mortality than are younger patients [18–20]. To decrease treatment-related mortality, risk factors and optimal dose intensity were assessed in older patients. Dose modification has been reported as a strategy for improving therapeutic outcomes [21]; however, the optimal dose for older patients with DLBCL remains unclear [22]. Although the determination of the optimal dose might decrease the risk of adverse drug events (including infection), RDI did not differ between the infection and non-infection groups in the present study. We could not conclude whether dose intensity affects the occurrence of infections events in the present study, and PS and the proportion of patients with a high NCCN-IPI were high in the infection group. A previous study showed that the proportion of infectious events was higher in a very high-risk age group (age: 60–75 years with PS = 4 or age >75 years) than that of in a standard-risk age group (age: 60–75 years with PS = ≤3 or age <60 years) groups [23]. The study revealed that multiple risk factors should be evaluated to prevent infectious events; however, the information on concomitant medications was limited. Therefore, we assessed whether NCCN-IPI and concomitant medications could be a risk factor for infection, and found that concomitant medications were associated with the occurrence of infection during chemotherapy in patients with DLBCL.

The occurrence rate of treatment-related adverse drug events in chemotherapy is higher than that associated with other treatments. In patients with metastatic cancer, high PS and polypharmacy are risk factors for hospitalization or an emergency room visit [24]. Therefore, it is necessary to assess the risk of polypharmacy in patients with cancer. Our results suggested that the number of concomitant medications was not associated with the occurrence of infection during chemotherapy. Since the number of concomitant medications is associated with comorbidity, we assessed the state of comorbidity by using CCI. Although a high CCI has been reported to be a risk factor for mortality in patients with DLBCL [10, 25], it was not the risk factor for infection events in our study. Interestingly, the use of benzodiazepines was found to be a risk factor for infection.

Two major screening tools recommended that older adults should avoid to use benzodiazepines [26, 27]. The peripheral benzodiazepine receptor is expressed in immune cells, and benzodiazepines have several effects on the immune system [28–31]. Diazepam serves as an immunomodulator, controlling undesired innate and adaptive immune responses [32]; furthermore, it has a notable suppressive effect on immune surveillance [33]. Additionally, sedative drug use results in impaired clearing of oral secretions because it inhibits the swallowing of saliva [34]. Further, relaxation of the lower esophageal sphincter by benzodiazepines might increase reflux events [35]. In the current study, the frequency of oral and gastrointestinal infection was higher than that of other infection sites, and the frequency of using antiulcer drugs in infection group was higher than that in the non-infection group. Thus, our results suggest that the impairment of oral secretions and the relaxation of the lower esophageal sphincter were associated with infections events. The patients with benzodiazepine use in our study had a low body surface area (S1 Table). Because sleep disorder is associated with a low skeletal muscle mass and poor physical performance [36], it was possibility that sarcopenia affected the immune system. Since we did not assess oral secretions, reflux events, and skeletal muscle mass, these are speculations for the mechanism for benzodiazepine-induced infection. However, benzodiazepine use might be a factor to consider for the occurrence of infection during chemotherapy in older patients with DLBCL.

Our study has several limitations. First, this was a single-center and retrospective study. The number of subjects and clinical parameters evaluated in our study were limited. To verify our results, the multicenter research and a large spontaneous reporting system (e.g., Japanese Adverse Drug Event Report database, FDA Adverse Event Reporting System) might be useful in the further study. Second, the patients received different chemotherapy regimens (i.e., R-CHOP, CHOP, and COP). Although the proportions of patients who received each of the chemotherapy regimens were not significantly different between the infection and non-infection groups, further studies should enroll patients who received the same chemotherapy regimen. Third, we could not come to a conclusion about the mechanism of benzodiazepine-induced infection. Oral secretions, reflux events, and skeletal muscle mass should be evaluated in further studies.

In conclusion, patients with DLBCL using benzodiazepines should be monitored for infection symptoms during chemotherapy.

## Supporting information

S1 Table Baseline characteristics of the patients in the benzodiazepines and non-benzodiazepines groups. CCI: Charlson Comorbidity Index, LDH: lactate dehydrogenase, NCCN-IPI: National Comprehensive Cancer Network-International Prognostic Index, R: rituximab, C: cyclophosphamide, H: adriamycin, O: vincristine, P: prednisolone, RDI: relative dose intensity, ^a^Fisher’s exact test, ^b^chi-square test.
(DOCX)

S1 Raw data (XLSX)
