# Supplementary material for: Use of benzodiazepines is the risk factor for infection in patients aged 80 years or older with diffuse large B-cell lymphoma: A single-institution retrospective study
Source: PLoS One. 2022 Jun 10;17(6):e0269362. doi: 10.1371/journal.pone.0269362 (PMC9187058; doi:10.1371/journal.pone.0269362)
Supplement: S1 Table — CCI: Charlson Comorbidity Index, LDH: lactate dehydrogenase, NCCN-IPI: National Comprehensive Cancer Network-International Prognostic Index, R: rituximab, C: cyclophosphamide, H: adriamycin, O: vincristine, P: prednisolone, RDI: relative dose intensity, aFisher’s exact test, bchi-square test. (DOCX) [file pone.0269362.s001.docx]

**Use of benzodiazepines is the risk factor for infection in patients aged 80 years or older with diffuse large B-cell lymphoma: a single‑institution retrospective study**

Anna Ogiso^1¶^, Tomohiro Mizuno^1¶*^, Kaori Ito^2^, Fumihiro Mizokami^3^, Akihiro Tomita^2^, Shigeki Yamada^1^

^1^ Department of Clinical Pharmacy, Fujita Health University School of Medicine, Toyoake, Japan

^2^ Department of Hematology, Fujita Health University School of Medicine, Toyoake, Japan

^3^ Department of Pharmacy, National Center for Geriatrics and Gerontology, Obu, Japan

^¶^These authors contributed equally to this work.

**^*^Corresponding author**

E-mail: [tomohiro.mizuno@fujita-hu.ac.jp](mailto:tomohiro.mizuno@fujita-hu.ac.jp) (TM)

**Supporting Information Table 1** Baseline characteristics of the patients in the benzodiazepines and non-benzodiazepines groups

| Baseline Characteristics | Benzodiazepines group (n=17) | Non-benzodiazepines group (n=48) | p value |
| --- | --- | --- | --- |
|  |  |  |  |
| Age, yrs (range) | 82.0 (80-90) | 83.0 (80-91) | 0.751 |
| Male, no (%) | 3 (17.6) | 20 (41.7) | 0.087^a^ |
| Body surface area (range) | 1.30 (1.06-1.67) | 1.45 (1.15-1.67) | 0.039 |
| Body mass index (range) | 20.1 (16.4-31.0) | 20.7 (15.5-33.4) | 0.465 |
| Performance status (range) | 2 (1-3) | 1.5 (0-4) | 0.268 |
| ≥ 2, no (%) | 12 (70.6) | 24 (50.0) | 0.142^b^ |
| Extranodal disease, no (%) | 7 (41.2) | 16 (33.3) | 0.569^a^ |
| Bone marrow infiltration, no (%) | 1 (5.88) | 8 (16.7) | 0.426^a^ |
| Central nervous system infiltration, no (%) | 0 (0) | 1 (2.08) | 1.000^a^ |
| Ann Arbor stage III-IV, no (%) | 9 (52.9) | 27 (56.3) | 1.000^a^ |
| Number of neutrophil, 10^^3^/μL (range) | 4.19 (1.86-12.5) | 3.74 (0.28-10.9) | 0.189 |
| Number of platelet, 10^^４^/μL (range) | 20.3 (13.0-37.2) | 19.2 (3.4-43.8) | 0.560 |
| Albumine, g/dＬ (range) | 3.4 (1.8-4.2) | 3.45 (2.1-5.2) | 0.284 |
| LDH ratio (range) | 1.27 (0.74-13.6) | 1.12 (0.60-4.84) | 0.923 |
| 0, no (%) | 7 (41.2) | 19 (39.6) | 0.541^b^ |
| 1, no (%) | 9 (52.9) | 22 (45.8) |  |
| 2, no (%) | 1 (5.88) | 7 (14.6) |  |
| NCCN-IPI | 6 (3-7) | 5 (3-8) | 0.523 |
| ≥ 6, no (%) | 9 (52.9) | 20 (41.7) | 0.571^a^ |
| CCI | 2 (2-8) | 3.5 (2-9) | 0.069 |
| ≥ 3, no (%) | 6 (35.3) | 28 (58.3) | 0.157^a^ |
| Number of concomitant medications | 6 (3-13) | 4 (0-15) | 0.093 |
| ≥ 6 medications, no (%) | 9 (52.9) | 20 (41.7) | 0.571^a^ |
| Chemotherapy regimen and RDI | | | |
| R-CHOP, no (%) | 7 (41.2) | 14 (29.2) | 0.938^b^ |
| CHOP, no (%) | 7 (41.2) | 20 (41.7) |  |
| COP, no (%) | 1 (5.88) | 4 (8.33) |  |
| R-COP, no (%) | 2 (11.8) | 7 (14.6) |  |
| CHP, no (%) | 0 (0) | 1 (2.08) |  |
| R-CHP, no (%) | 0 (0) | 1 (2.08) |  |
| R-HOP, no (%) | 0 (0) | 1 (2.08) |  |
| Rituximab RDI, % (range) | 100 (0-105) | 98.3 (0-106) | 0.988 |
| Cyclophosphamide RDI, % (range) | 72.4 (50.6-83.3) | 68.5 (0-81.0) | 0.199 |
| Doxorubicin RDI, % (range) | 59.9 (0-83.3) | 59.9 (0-80.2) | 0.356 |
| Vincristine RDI, % (range) | 64.6 (42.9-73.6) | 68.6 (0-100) | 0.893 |
| Prednisolone, mg (range) | 50 (10-65) | 52 (0-100) | 0.236 |

CCI: Charlson Comorbidity Index, LDH: lactate dehydrogenase, NCCN-IPI: National Comprehensive Cancer Network-International Prognostic Index, R: rituximab, C: cyclophosphamide, H: adriamycin, O: vincristine, P: prednisolone, RDI: relative dose intensity, ^a^Fisher's exact test, ^b^chi-square test
